# On the origin of carbon dioxide released from rewetted soils

**DOI:** 10.1016/j.soilbio.2016.06.032

**Published:** 2016-10

**Authors:** F.C. Fraser, R. Corstanje, L.K. Deeks, J.A. Harris, M. Pawlett, L.C. Todman, A.P. Whitmore, K. Ritz

**Affiliations:** a School of Energy, Environment, and Agrifood, Cranfield University, Bedford, UK; b Rothamsted Research, Harpenden, UK; c School of Biosciences, University of Nottingham, Nottingham, UK

**Keywords:** Birch effect, Dry:wet cycles, CO_2_ flux, Extracellular oxidative metabolism, Soil sterilisation

## Abstract

When dry soils are rewetted a pulse of CO_2_ is invariably released, and whilst this phenomenon has been studied for decades, the precise origins of this CO_2_ remain obscure. We postulate that it could be of chemical (i.e. via abiotic pathways), biochemical (via free enzymes) or biological (via intact cells) origin. To elucidate the relative contributions of the pathways, dry soils were either sterilised (double autoclaving) or treated with solutions of inhibitors (15% trichloroacetic acid or 1% silver nitrate) targeting the different modes. The rapidity of CO_2_ release from the soils after the drying:rewetting (DRW) cycle was remarkable, with maximal rates of evolution within 6 min, and 41% of the total efflux over 96 h released within the first 24 h. The complete cessation of CO_2_ eflux following sterilisation showed there was no abiotic (dissolution of carbonates) contribution to the CO_2_ release on rewetting, and clear evidence for an organismal or biochemical basis to the flush. Rehydration in the presence of inhibitors indicated that there were approximately equal contributions from biochemical (outside membranes) and organismal (inside membranes) sources within the first 24 h after rewetting. This suggests that some of the flux was derived from microbial respiration, whilst the remainder was a consequence of enzyme activity, possibly through remnant respiratory pathways in the debris of dead cells.

## Highlights

• Soils rewetted after drying exhibit an extremely rapid flux of CO_2_, peak efflux occurs in less than 6 min.
• Such CO_2_ fluxes were prevented by autoclaving, suggesting an intrinsically biochemical or organismal origin to the source.
• Strong evidence for an extracellular oxidative pathway contributing to such CO_2_ fluxes was found.

Rewetting of a dry soil invariably causes a large flux of carbon dioxide (CO_2_) to be rapidly released, which is sometimes referred to as the Birch effect (Birch, 1958, Birch, 1960). This phenomenon has been observed both in laboratory incubations (Kieft et al., 1987, Unger et al., 2010, Shi and Marschner, 2014) and in field circumstances using closed chambers (Yan et al., 2014) or eddy covariance towers (Xu et al., 2004). These fluxes have been observed across a wide range of ecotypes (Jarvis et al., 2007, Thomas and Hoon, 2010, Sugihara et al., 2015), but are particularly significant in dryland and Mediterranean ecosystems where they can make up a significant proportion of soil C-emissions (Lee et al., 2004, Hunt et al., 2004, Brito et al., 2013). These drying:rewetting (DRW) induced CO_2_ efflux events can even significantly reduce the annual net C gain in Mediterranean forests (Jarvis et al., 2007).

Several theories have been proposed to explain this phenomenon including: (i) the exposure of physically-protected organic matter to microbial metabolism via aggregate dispersion on rewetting (Denef et al., 2001, Wu and Brookes, 2005, Xiang et al., 2008); (ii) microbial necromass increasing the supply of readily assimilable substrate to the surviving microbial populations (Kieft et al., 1987, Van Gestel et al., 1992, Blazewicz et al., 2013); (iii) increases in the supply of labile organic matter due to the rapid release, on rewetting, of intra-cellular solutes previously concentrated within microbial cells to maintain osmotic balance in response to dehydration (Halverson et al., 2000, Warren, 2014); and (iv) a supply of labile organic C is built up during the dry period prior to rewetting and subsequently quickly metabolised on rewetting. There is a known uncoupling of rates of CO_2_ efflux and detectable microbial growth rates after a DRW cycle (Iovieno and Bååth, 2008, Meisner et al., 2015) and microbial populations in such circumstances show little change in their net size (Fierer and Schimel, 2002). However, recent work by Blazewicz et al. (2013) show that despite their unchanging size these populations turnover rapidly in response to a DRW cycle. They also suggest that more cellular derived organic-C is available in soil samples than is turned over in the initial phases after rewetting. This organic-C will contain cellular material including constituents of enzymatic pathways – remnant respiratory pathways – with the potential to carry out reactions leading to CO_2_ efflux. Thus it is possible that CO_2_ release from re-wetted soils is not exclusively derived from respiration pathways occurring in intact microbes. There are also reports of over-estimation of soil respiration rates due to contributions of CO_2_ from dissolution of soil carbonates; however, reports are inconsistent and range from 1 to 2% up to 74% of CO_2_ efflux from soil being attributed to carbonate dissolution (Biasi et al., 2008, Ramnarine et al., 2012, Schindlbacher et al., 2015). It is as yet unclear how the DRW process may affect carbonate dissolution from soils although Tamir et al. (2011) found that in highly calcareous soils the rate of inorganic CO_2_ production was lower in drier samples. However, it is also known that increases in soil OM content can alter the balance of pH, as a result of increased nitrification rates, leading to increase dissolution of carbonates (Tamir et al., 2013). As such an increase in available OM as a result of any of the 4 processes described above (aggregate dispersion, increased necromass, release of intracellular-solutes, or accumulation of labile organic matter) could potentially lead to this phenomenon on rewetting, and an abiotic route to CO_2_ production must also be considered.

**Fig. 1:**
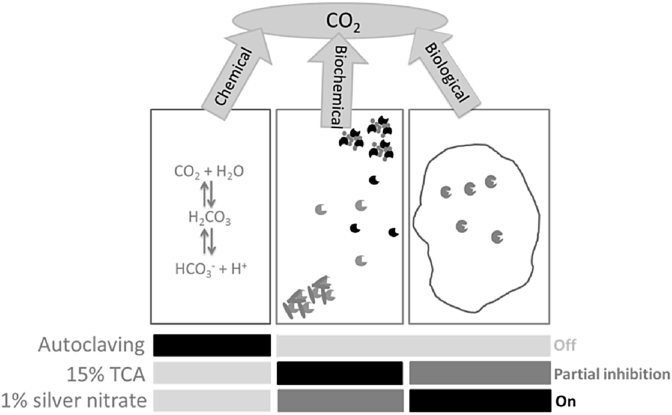
Three potential sources of CO_2_ to account for the flush on rewetting of dry soils and the treatments used to identify the respective contributions of these. Light grey bars in lower panel indicates which potential sources of CO_2_ are uninhibited by each treatment, mid-grey shows which sources are potentially inhibited, and dark grey shows those that are ‘switched off’ by the different treatments.

On this basis we posit that there are three potential sources of CO_2_, all of which could contribute to the efflux on rewetting: (i) *abiotic* via carbonate dissolution (Shanhun et al., 2012); (ii) *biochemical*, involving the release of CO_2_ from organic matter outside cell membranes and mediated by free or residually-bound enzymes (Maire et al., 2013) (Blankinship et al., 2014); (iii) *organismal*, i.e. microbial respiration via the Krebs cycle carried out within intact organelles or cells (Fig. 1). One potential way to determine the relative contribution of these sources is to probe the phenomenon in soils treated in various ways to block certain of the pathways involved, such as via complete sterilisation (i.e. any form of biochemical or organismal pathway), or to spike the rehydration water with various forms of metabolic inhibitors (i.e. to distinguish biochemical from organismal). We hypothesised that i) the majority of CO_2_ released is derived from an organismal source, and hence that CO_2_ efflux upon rehydration would be curtailed where organismal pathways were blocked and ii) there would be no significant contribution to the total CO_2_ efflux of CO_2_ from an abiotic source.

Soils were collected from the top 15 cm of 4 long-term grassland sites in May 2015 (soil parameters shown in Table 1); all soils were sieved to pass a 2 mm mesh, adjusted to 45% water holding capacity (WHC) and pre-incubated at 25 °C for 7 days. Aliquots of the soils (1 g; 3 replicates of each soil) were then exposed to 4 DRW cycles over 28 days, where each cycle consisted of 3 days drying followed by rewetting to 45% WHC using sterile, deionised water. Drying was standardised by locating the soils in a sealed container in the presence of silica gel. Aliquots of 1.0 g of soil were adopted in order to ensure that penetration of water throughout the soil volume would be rapid. The time-course of CO_2_ evolution at 6-min intervals following rewetting was determined independently for each replicate using an automated multi-channel conductimetric respirometer (RABIT, Don Whitley, Shipley, UK; (Butler et al., 2012), for 5 days. To account for any background variation in CO_2_ efflux blanks were run alongside soil samples; this involved measuring the signal from empty, sealed cells.

Another set of three replicates was subjected to a further range of treatments, *viz*. (i) ‘Live controls’ - involving no sterilisation, DRW as described above; (ii) ‘Moist controls’ – also unsterilized but with 0.2 mL sterile, deionised water added prior to exposure to DRW – this is a procedural control to account for the fact that liquid was added to the sample prior to drying as described above; (iii) ‘Autoclaved’, where samples were autoclaved twice at 121 °C at 3.1 bar for 20 min with a 24 h pause between (Systec 3150 EL, Linden, Germany); (iv) ‘TCA’, with 0.2 mL of 15% trichloroacetic acid (TCA) addition; (v) ‘AgNO_3_’, with 0.2 mL of 1% silver nitrate addition. All amendments and autoclaving were carried out prior to the DRW process described above. The rationale for these treatments (Fig. 1) is that autoclaving would prevent all organismal or biochemical activity by denaturing all proteins – in this circumstance any CO_2_ produced would be via abiotic pathways. TCA (15%) would precipitate proteins, including extracellular enzymes (Ladd and Butler, 1972) and as such remove any biochemical source of CO_2_. The mechanism of protein precipitation by TCA is unclear but is likely to be due to protein unfolding (Rajalingam et al., 2009) and as such may also affect microbial membranes. AgNO_3_ is a known antiseptic and so kills microbes; the precise mode of action is surprisingly poorly understood but the Ag^+^ ions are known to cause physical damage to cells and DNA – separation of cytoplasmic membranes from cell walls and condensing of DNA in both *Escherichia coli* and *Staphylococcus aureus* (Feng et al., 2000). Silver and other heavy metals are also known to bind to thiol groups in proteins resulting in their inactivation (Liau et al., 1997). They also interfere with intra-cellular processes and membranes/cell walls therefore AgNO_3_ may also affect some extracellular enzymes (e.g. thiol-proteases). This treatment is designed to primarily inhibit the organismal pathway but is likely to have a lesser effect on biochemical mechanisms – i.e. extracellular enzymes (Fig. 1). Whilst the extent to which these inhibitors operate exclusively on these pathways is unknown (and may be impossible to precisely establish), the rationale is that they will be at least partly informative. However, autoclaving twice unequivocally sterilises soil.

**Fig. 2:**
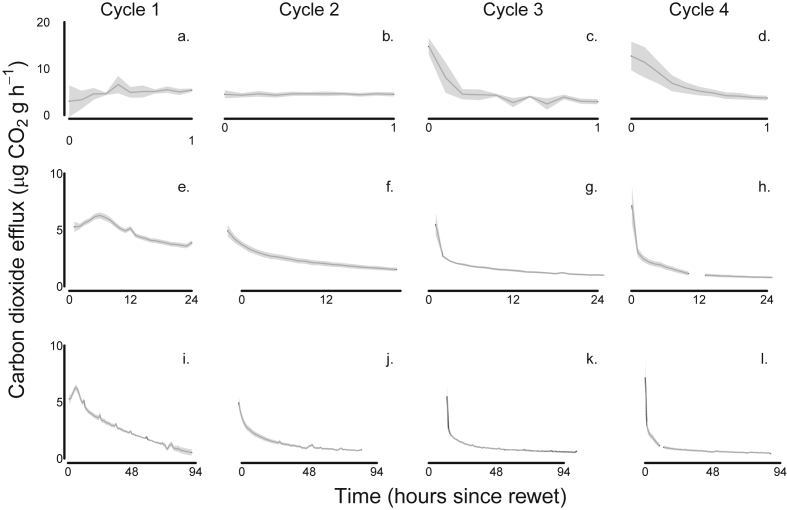
CO_2_ release profiles from unsterilized grassland soil exposed to 4 repeated DRW events (Cycles 1–4); (a–d) CO_2_ release measured at 6 min intervals in the first hour after rewetting, (e–h) hourly CO_2_ release over the first 24 h after rewetting, (I – l) hourly CO_2_ release over the entire 94 h wet period. Means (n = 3) indicated by black line surrounded by confidence bands of ±1 standard error.

The rapidity of CO_2_ release from the soils after the DRW cycle was remarkable, in that we detected maximal rates of evolution after 6 min, and never captured the actual peak as such, only a downward trend from a presumed peak (Fig. 2). Within the first hour following wet-up an average of 5% of the total CO_2_ efflux over 96 h was observed and of this approximately 24% occurred within the first 12 min (Fig. 2a–d). Of the total CO_2_ efflux measured over 96 h after rewetting, an average of 41% was measured in the first 24 h (Fig. 2e–h); this consistency of effect with – where the same proportion of CO_2_ was measured in the first 24 h after each of a series of rewetting events - was also observed by Birch (1958).

**Fig. 3:**
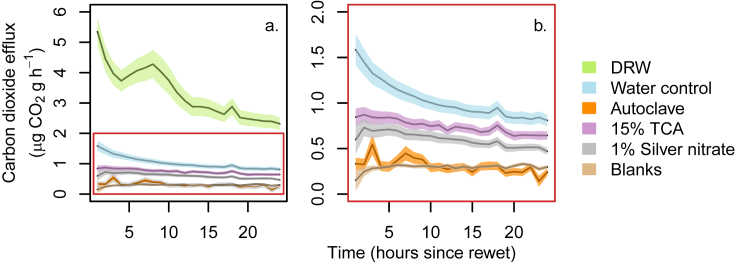
CO_2_ efflux rates following rewetting of a dry soil with various solutions; (a) live soil (green) exposed to a DRW cycle compared to all other treatments including a moist control (blue), area outlined in red is shown in greater detail in (b); (b) amplification of y-axis from (a), i.e. CO_2_ efflux following a DRW cycle from the moist control (blue), blanks (no soil - brown), autoclaved (orange), 15% TCA (purple) and 1% silver nitrate (grey) treated soils. Lines show mean rates of CO_2_ efflux (n = 12 (3 reps each of 4 soils)) surrounded by confidence bands of ±1 standard error.

A large difference in CO_2_ release on rewetting between the wet control and the standard response to DRW was manifest (Fig. 3a). This is likely because the 3-day drying period resulted in different amounts of moisture loss between treatments; those exposed to the prescribed DRW cycle lost 34% of their mass on average over the 3 days of drying, however, the moist controls lost only 16% of their mass on average. This shows that soil dried to a greater extent will give a larger flush of CO_2_ on rewetting than a sample of the same soil dried less severely (Kieft et al., 1987, Fierer and Schimel, 2002, Unger et al., 2010, Meisner et al., 2015). Those samples treated with 15% TCA and 1% AgNO_3_ dried to a greater extent over 3 days than the moist controls (21 and 28% mass loss respectively) and those that were autoclaved lost 45% of their mass on average. Despite these large differences in moisture loss between the moist controls and the inhibitor treated samples (both TCA and AgNO_3_) the effect of moisture loss on total CO_2_ efflux was found to be non-significant using an analysis of covariance (ANCOVA; p = 0.71), nor was there a significant interaction between inhibitor treatment and moisture loss (p = 0.25). As such, the main effect of inhibitor treatment can be interpreted directly.

Hereafter, responses of inhibitor-treated samples to DRW are compared to that of the moist controls (Fig. 3b). Autoclaving effectively ‘switched off’ CO_2_ production after a rewetting event (total CO_2_ efflux over 24 h was significantly different between water controls and autoclaved samples and autoclaved totals were not significantly different from blanks (p = 0.01, p = 0.99 respectively, Fig. 3). A preliminary experiment using soil with higher CaCO_3_ contents (0.93% compared to 0.48% on average for soils listed in Table 1) showed the same lack of activity after autoclaving and a DRW event (data not presented). These results show that there was effectively no chemical contribution to the CO_2_ flush observed after rewetting in these soils. This is in contrast to observations made in some calcareous, arid soils where CO_2_ derived from inorganic–C has been observed to account for 30–75% of the total soil CO_2_ efflux (Tamir et al., 2011, Shanhun et al., 2012). As previously stated, these observations have also been made in temperate soils but results are scarce and inconsistent with ranges of 1–2% (Schindlbacher et al., 2015), to 50% (Biasi et al., 2008) all the way up to 74% (Ramnarine et al., 2012) of the total CO_2_ flux attributable to inorganic C sources. Notably, none of these studies examined the response to a DRW event although Biasi et al. (2008) noted an effect of water addition in the laboratory. The effect of autoclaving observed in our study is therefore strong evidence for an organismal and/or biochemical origin for the evolved CO_2_

Treating soils with either 15% TCA or 1% AgNO_3_ substantially reduced but did not eliminate CO_2_ production, compared to the moist control, following a DRW event (Fig. 3b). Inhibition of CO_2_ evolution by AgNO3 was greater than by TCA for the latter half of the measurement period (Fig. 3), although the accumulated total release was not statistically significant in the case of these two inhibitors (p = 0.98). This suggests that a greater portion of the CO_2_ measured after a DRW event is derived from the organismal pathway. This effect appeared to increase over time with the amount of CO_2_ produced hourly by AgNO_3_ treated soils decreasing more rapidly over the first 24 h than it did for TCA treated soils this is exemplified by the increasing gap between the confidence bands for AgNO_3_ and TCA treated soils after approximately 13 h of incubation in Fig. 3. It is commonly assumed that the majority of CO_2_ measured after a DRW event is derived from the organismal pathway, and the effect of AgNO_3_ would certainly suggest this. There was also a substantial reduction in CO_2_, compared to the moist control, due to the addition of TCA, which suggests that an additional contribution to the CO_2_ flux after the DRW event was via the biochemical route. This is consistent with the findings of Maire et al. (2013) who report a 16–48% contribution of an extracellular oxidative metabolism pathway, termed ‘EXOMET’, to soil CO_2_ flux. Blankinship et al. (2014) found only a 26–47% reduction in CO_2_ emission from intermediates in the TCA cycle after sterilisation suggesting that these enzymes are still active when cells are dead but not completely dispersed, again noting that neither of these two studies were in response to DRW events. It is known that many enzymes are stable in the soil environment on a long term basis (Burns et al., 2013). Such stability is generally achieved by adsorption onto soil colloids or incorporation with humic complexes (Nannipieri et al., 1996). The effects of adsorption or humic complexing can include inhibition and steric hindrance which can cause a reduction in potential activity of this sizeable enzyme pool by up to 90% (Quiquampoix et al., 2002). If even a small proportion of these enzymes were to be brought into solution after rewetting this could have a large effect on the levels of activity in soils (Stursova and Sinsabaugh, 2008). Significant increases in rates of enzyme activity have been recorded in soils exposed to DRW both during laboratory preparation (Kandeler and Gerber, 1988) and as a result of environmental conditions (Hinojosa et al., 2004) suggesting that portions of the adsorbed enzyme pool are solubilised by the process of rewetting after drying increasing the potential for a biochemically driven response in DRW soils.

Our results demonstrate the apparent immediacy of the Birch effect, and go some way to explaining the pathways by which the CO_2_ is evolved, *viz*. primarily organismal but with a potentially large contribution from the biochemical pathways. We note that for our experiments, these are roughly equivalent in magnitude. Thus we reject the hypothesis that the origin of the CO_2_ released following rehydration is predominantly organismal. We have shown that in these temperate soils, unlike in more calcareous, arid systems, there is no contribution of carbonate dissolution even when the intrinsic concentration of CaCO_3_ is high. This means that this effectively instantaneous release of CO_2_ is governed by the soil biota. We have shown evidence that not only are intact microbial cells apparently capable of reinstating their high rates of respiration within minutes following rehydration after 3 days of drying, but also that there is a potentially extensive contribution of CO_2_ from remnant enzymatic pathways outside of cell membranes.

## Figures and Tables

**Table 1:** Locations from which soils sampled (latitude and longitude) and associated basic properties.

Soil	Latitude: Longitude	Sand (%)	Silt (%)	Clay (%)	N (%)	C (%)	C:N	pH	Water-holding capacity (ml g^−1^)	Loss on ignition (g g soil^−1^)	Microbial biomass C(μg g^−1^)	Inorganic C-content (%)
A	52.4245°N: −4.0652°W	7.5	53.9	38.6	0.7	7.6	10.8	5.5	0.98	0.150	2330	0.18
B	53.2222°N: −4.0132°W	28.7	41.8	29.4	0.8	9.5	11.4	5.1	0.10	0.169	1699	0.74
C	53.0412°N: −4.0445°W	34.8	48.9	16.3	0.6	6.1	10.8	5.8	0.91	0.127	1407	0.74
D	52.9988°N: −4.4290°W	75.3	24.4	0.3	0.3	3.4	10.7	5.6	0.70	0.065	739	0.26
